# Supplementary material for: Silver Birch (Betula pendula Roth.) on Dry Mineral Rather than on Deep Peat Soils Is More Dependent on Frozen Conditions in Terms of Wind Damage in the Eastern Baltic Region
Source: Plants (Basel). 2022 Apr 26;11(9):1174. doi: 10.3390/plants11091174 (PMC9104462; doi:10.3390/plants11091174)
Supplement: Supplementary file 1 [file plants-11-01174-s001.zip › plants-1678154-supplementary.pdf]

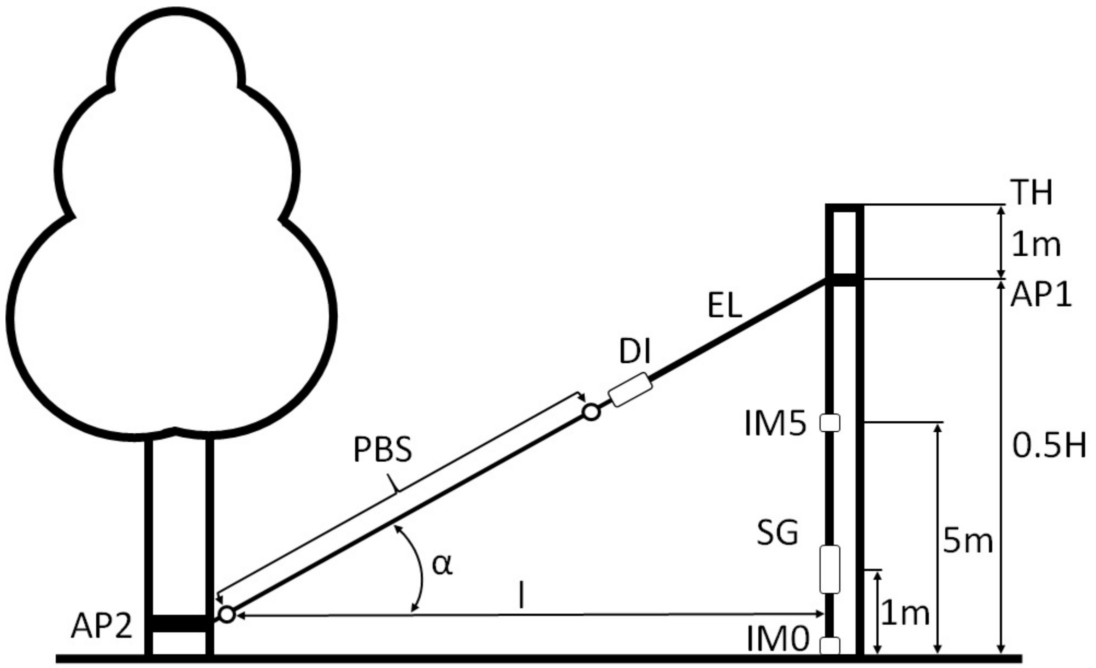

Figure S1. A scheme of the setup of static tree-pulling test. AP1, AP2–upper and base anchor points of pulling line, respectively; PBS–pulley block system; EL–extension line; DI–dynamometer; α–angle between the pulling line and ground; l–distance from the anchoring tree; IM0, IM5– inclinometers at the stem base and at 5 m height, respectively; SG–strain gauge; 0.5H–half tree height; TH–topping height.

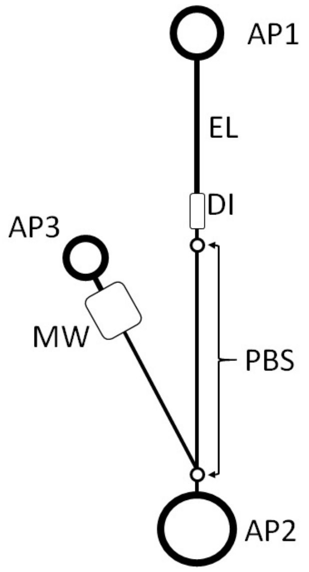

Figure S2. The location of motor winch during the static tree-pulling test. AP1, AP2 and AP3–anchoring points; MW–motor-winch; PBS–pulley block system; EL– extension line; DI– dynamometer.
